# Exemplary multiplex bisulfite amplicon data used to demonstrate the utility of Methpat

**DOI:** 10.1186/s13742-015-0098-x

**Published:** 2015-11-26

**Authors:** Nicholas C. Wong, Bernard J. Pope, Ida Candiloro, Darren Korbie, Matt Trau, Stephen Q. Wong, Thomas Mikeska, Bryce J. W. van Denderen, Erik W. Thompson, Stefanie Eggers, Stephen R. Doyle, Alexander Dobrovic

**Affiliations:** 1 Translational Genomics and Epigenomics Laboratory, Olivia Newton-John Cancer Research Institute, Heidelberg, VIC 3084 Australia; 2 Murdoch Childrens Research Institute, The Royal Children’s Hospital, Parkville, VIC 3052 Australia; 3 Department of Paediatrics, The University of Melbourne, Parkville, VIC 3052 Australia; 4 Victorian Life Sciences Computation Initiative (VLSCI), The University of Melbourne, Parkville, VIC 3052 Australia; 5 Department of Computing and Information Systems, The University of Melbourne, Parkville, VIC 3052 Australia; 6 Department of Microbiology and Immunology, The University of Melbourne at the Peter Doherty Institute for Infection and Immunity, Parkville, VIC 3010 Australia; 7 Department of Pathology, The University of Melbourne, Parkville, VIC 3052 Australia; 8 Centre for Personalised NanoMedicine, Australian Institute of Bioengineering and Nanotechnology, The University of Queensland, Brisbane, QLD 4072 Australia; 9 School of Chemistry and Molecular Biosciences, University of Queensland, Brisbane, QLD 4072 Australia; 10 Division of Cancer Research, Peter MacCallum Cancer Centre, East Melbourne, VIC 3002 Australia; 11 St Vincent’s Institute of Medical Research, 9 Princes Street, Fitzroy, 3065 Australia; 12 Institute of Health and Biomedical Innovation and School of Biomedical Sciences, Queensland University of Technology, Brisbane, QLD 4059 Australia; 13 Department of Animal, Plant and Soil Sciences, La Trobe University, Bundoora, VIC 3086 Australia; 14 Division of Cancer Medicine, La Trobe University, Bundoora, VIC 3086 Australia; 15 School of Cancer Medicine, La Trobe University, Bundoora, VIC 3084 Australia; 16 Molecular Pathology Research and Development Laboratory, Department of Pathology, Peter MacCallum Cancer Centre, East Melbourne, VIC 3002 Australia; 17 Present Addresses: Pacific Edge Biotechnology Ltd, Dunedin, Otago 9016 New Zealand; 18 Translational Research Laboratory, Division of Cancer Research, Peter MacCallum Cancer Centre, East Melbourne, VIC 3002 Australia

**Keywords:** DNA methylation, Bisulfite sequencing, PCR, Visualization, Epigenetics, Cancer, Epialleles

## Abstract

**Background:**

DNA methylation is a complex epigenetic marker that can be analyzed using a wide variety of methods. Interpretation and visualization of DNA methylation data can mask complexity in terms of methylation status at each CpG site, cellular heterogeneity of samples and allelic DNA methylation patterns within a given DNA strand. Bisulfite sequencing is considered the gold standard, but visualization of massively parallel sequencing results remains a significant challenge.

**Findings:**

We created a program called Methpat that facilitates visualization and interpretation of bisulfite sequencing data generated by massively parallel sequencing. To demonstrate this, we performed multiplex PCR that targeted 48 regions of interest across 86 human samples. The regions selected included known gene promoters associated with cancer, repetitive elements, known imprinted regions and mitochondrial genomic sequences. We interrogated a range of samples including human cell lines, primary tumours and primary tissue samples. Methpat generates two forms of output: a tab-delimited text file for each sample that summarizes DNA methylation patterns and their read counts for each amplicon, and a HTML file that summarizes this data visually. Methpat can be used with publicly available whole genome bisulfite sequencing and reduced representation bisulfite sequencing datasets with sufficient read depths.

**Conclusions:**

Using Methpat, complex DNA methylation data derived from massively parallel sequencing can be summarized and visualized for biological interpretation. By accounting for allelic DNA methylation states and their abundance in a sample, Methpat can unmask the complexity of DNA methylation and yield further biological insight in existing datasets.

## Data description

DNA methylation can be analyzed using a wide range of methods [1], with bisulfite sequencing considered the current gold standard. Current technologies such as whole genome bisulfite sequencing (WGBS) and reduced representation bisulfite sequencing (RRBS) provide unprecedented detail of methylation patterns throughout the genome, but the complexity of DNA methylation patterns is masked when simple summary metrics are used. For example, most studies of DNA methylation rationalize levels to a percentage value, which typically masks allelic patterns when interpreting the data. We have developed Methpat, a tool that summarizes and visualizes complex DNA methylation data collected by massively parallel sequencing of bisulfite DNA [2]. Using this tool, the DNA methylation state of individual CpG sites and the abundance of allelic patterns can be visualized [3]. Furthermore, by measuring the abundance of allelic DNA methylation patterns, cellular heterogeneity in methylation patterns can now be explored [4].

The utility of Methpat was demonstrated by measuring DNA methylation in 86 samples (Table 1) across 48 regions of interest (Table 2). This was achieved by using multiplex PCR on bisulfite converted DNA followed by massively parallel sequencing using an Illumina MiSeq Sequencing platform with v3 chemistry. Each sample was indexed and pooled at equimolar concentrations into a single library pool for sequencing. Data has been deposited into GEO with reference identifiers GSE67856 [5] and GSE71804 [6]. A panel of breast cancer cell lines treated with epidermal growth factor and transforming growth factor beta were also analyzed in parallel [7].

**Table 1:** Human Samples used in this study

Sample Name	Description	GEO Accession
293	HEK-293 embryonic kidney cell line. ATCC CRL1573	GSE67856
40424	Normal fibroblast cell line	GSE67856
910046	Normal fibroblast cell line	GSE67856
12A-CD19	Normal Fluorescent Activated Cell Sorted (FACS) CD19 positive bone marrow cells from individual 12A	GSE67856
12A-CD33	Normal Fluorescent Activated Cell Sorted (FACS) CD33 positive bone marrow cells from individual 12A	GSE67856
12A-CD34	Normal Fluorescent Activated Cell Sorted (FACS) CD34 positive bone marrow cells from individual 12A	GSE67856
12A-CD45	Normal Fluorescent Activated Cell Sorted (FACS) CD45 positive bone marrow cells from individual 12A	GSE67856
6-MDA453	MDA-MB-453 metastatic breast cancer cell line. ATCC HTB-131	GSE67856
6C-CD19	Normal Fluorescent Activated Cell Sorted (FACS) CD19 positive bone marrow cells from individual 6C	GSE67856
6C-CD33	Normal Fluorescent Activated Cell Sorted (FACS) CD33 positive bone marrow cells from individual 6C	GSE67856
6C-CD34	Normal Fluorescent Activated Cell Sorted (FACS) CD34 positive bone marrow cells from individual 6C	GSE67856
6C-CD45	Normal Fluorescent Activated Cell Sorted (FACS) CD45 positive bone marrow cells from individual 6C	GSE67856
9A-CD19	Normal Fluorescent Activated Cell Sorted (FACS) CD19 positive bone marrow cells from individual 9A	GSE67856
9A-CD33	Normal Fluorescent Activated Cell Sorted (FACS) CD33 positive bone marrow cells from individual 9A	GSE67856
9A-CD34	Normal Fluorescent Activated Cell Sorted (FACS) CD34 positive bone marrow cells from individual 9A	GSE67856
9A-CD45	Normal Fluorescent Activated Cell Sorted (FACS) CD45 positive bone marrow cells from individual 9A	GSE67856
9A-Whole-Blood	Whole blood sample from individual 9A	GSE67856
BRL	Normal lymphoblast cell line.	GSE67856
CaCo	Caco2 Colon cancer cell line. ATCC HTB37	GSE67856
DG75	Lymphoblast cancer cell line. ATCC CRL-2625	GSE67856
EKVX	Cancer Cell Line	GSE67856
HELA	Cancer cell line. ATCC CCL-2	GSE67856
HEPG2	Liver cancer cell line. ATCC HB-8065	GSE67856
HT1080	Cancer cell line. ATCC CCL121	GSE67856
HTB22-Col	MCF7 breast cancer cell line. ATCC HTB22	GSE67856
JWL	Normal lymphoblast cell line.	GSE67856
K562	CML cancer cell line. ATCC CCL-243	GSE67856
Sample29	Cell Line	GSE71804
MB231BAG	Breast cancer cell line. ATCC HTB-26	GSE67856
MCF7	Breast cancer cell line. ATCC HTB22	GSE67856
NALM6	Leukaemia cell line. ACC 128	GSE67856
NCCIT	Embryonic carcinoma cell line. ATCC CRL-2073	GSE67856
OVCAR8	Cancer cell line	GSE67856
SKNAS	Neuroblastoma cancer cell line. ATCC CRL2137	GSE67856
U231	Cancer cell line	GSE67856
Sample1	Human normal colon tissue	GSE71804
Sample2	Human colon tumor	GSE71804
Sample3	Human normal colon tissue	GSE71804
Sample4	Human colon tumor	GSE71804
Sample5	Human normal colon tissue	GSE71804
Sample6	Human colon tumor	GSE71804
Sample7	Human normal colon tissue	GSE71804
Sample8	Human colon tumor	GSE71804
Sample9	Human normal colon tissue	GSE71804
Sample10	Human colon tumor	GSE71804
Sample11	Human normal colon tissue	GSE71804
Sample12	Human colon tumor	GSE71804
Sample13	Pooled human cancer and blood cell DNA	GSE71804
Sample14	Pooled human cancer and blood cell DNA	GSE71804
Sample15	Pooled human cancer and blood cell DNA	GSE71804
Sample16	Pooled human cancer and blood cell DNA	GSE71804
Sample17	Pooled human cancer and blood cell DNA	GSE71804
Sample18	Pooled human cancer and blood cell DNA	GSE71804
Sample19	Artificially methylated human DNA	GSE71804
Sample20	Artificially methylated human DNA	GSE71804
Sample21	Artificially methylated human DNA	GSE71804
Sample22	Artificially methylated human DNA	GSE71804
Sample23	Artificially methylated human DNA	GSE71804
Sample24	Artificially methylated human DNA	GSE71804
Sample25	Human leukemia cell line	GSE71804
Sample26	Human leukemia cell line	GSE71804
Sample27	Human leukemia cell line	GSE71804
Sample28	Human leukemia cell line	GSE71804
468-C1-3-9_S40	MDA-468 cell line, control 1	GSE71804
468-C2-3-9_S48	MDA-468 cell line, control 2	GSE71804
468-S1-3-9_S56	MDA-468 cell line + EGF 1	GSE71804
468-S2-3-9_S64	MDA-468 cell line + EGF 2	GSE71804
ET-C1-3-9_S71	PMC42-ET cell line, control 1	GSE71804
ET-C2-3-9_S79	PMC42-ET cell line, control 2	GSE71804
ET-S1-3-9_S87	PMC42-ET cell line, +EGF 1	GSE71804
ET-S2-3-9_S95	PMC42-ET cell line, +EGF 2	GSE71804
LA-C1-3-9_S8	PMC42-LA cell line, control 1	GSE71804
LA-C3-3-9_S16	PMC42-LA cell line, control 2	GSE71804
LA-S1-3-9_S24	PMC42-LA cell line, +EGF 1	GSE71804
LA-S2-3-9_S32	PMC42-LA cell line, +EGF 2	GSE71804
PMC42ET-72-C_S31	PMC42-ET cell line, control 72 h	GSE71804
PMC42ET-72 h-EGF_S39	PMC42-ET cell line, +EGF 72 h	GSE71804
PMC42ET-9d-C_S47	PMC42-ET cell line, control 9 days	GSE71804
PMC42ET-9d-EGF_S55	PMC42-ET cell line, +EGF 9 days	GSE71804
PMC42ET-9d-TGFb_S63	PMC42-ET cell line, +TGFb 9 days	GSE71804
PMC42LA-72 h-C_S86	PMC42-LA cell line, control 72 h	GSE71804
PMC42LA-72 h-EGF_S94	PMC42-LA cell line, +EGF 72 h	GSE71804
PMC42LA-9d-C_S7	PMC42-LA cell line, control 9 days	GSE71804
PMC42LA-9d-EGF_S15	PMC42-LA cell line, +EGF 9 days	GSE71804
PMC42LA-9d-TGFb_S23	PMC42-LA cell line, +TGFb 9 days	GSE71804

**Table 2:** Bisulfite PCR primers used in this study

Primer name	Primer sequence	Primer Tm	Genomic location (hg38)
mandatory01_plus_F	TCGTCGGCAGCGTCAGATGTGTATAAGAGACAGGAGAAGTTTGGTYGTTGYGTTTTTAT	60.1–62.9	
mandatory01_plus_R	GTCTCGTGGGCTCGGAGATGTGTATAAGAGACAGRAAACCRCTCRCRAAATACCCTA	57.6–64.6	chr4:154710460-154710544
mandatory02_plus_F	TCGTCGGCAGCGTCAGATGTGTATAAGAGACAGTAGYGGAGTTTAAGGGTTAGTGT	59.2–60.9	
mandatory02_plus_R	GTCTCGTGGGCTCGGAGATGTGTATAAGAGACAGAACRAAACRCACRTACRTATATTTATA	56.3–62.1	chr1:110052409-110052486
mandatory03_plus_F	TCGTCGGCAGCGTCAGATGTGTATAAGAGACAGTGTTTGTTAGTTAGTTTTAGGTTTTTTAAT	59.8	
mandatory03_plus_R	GTCTCGTGGGCTCGGAGATGTGTATAAGAGACAGCCTACCAAATTTCTATTACAAACCAAA	60.8	chr4:7526639-7526703
mandatory04_plus_F	TCGTCGGCAGCGTCAGATGTGTATAAGAGACAGGATTTGGTTTYGAGAGTTTGGATTTT	60.1–61.7	
mandatory04_plus_R	GTCTCGTGGGCTCGGAGATGTGTATAAGAGACAGAAAAACCRCACACCTAAACACTTAAA	60.1–61.7	chr2:164593225-164593299
mandatory05_plus_F	TCGTCGGCAGCGTCAGATGTGTATAAGAGACAGGGGAATTTTGAGATTTTTAAAAGTTTTTTT	59.8	
mandatory05_plus_R	GTCTCGTGGGCTCGGAGATGTGTATAAGAGACAGATAAAAACAACAAATACCACTTCCTAAA	59.9	chr2:9518296-9518358
mandatory06_plus_F	TCGTCGGCAGCGTCAGATGTGTATAAGAGACAGTTGYGTYGATTTTGGTTTTGGTTAT	57.6–60.9	
mandatory06_plus_R	GTCTCGTGGGCTCGGAGATGTGTATAAGAGACAGCRACCCCTCCCAAATCCTAAAA	60.1–62.1	chr17:80709100-80709203
mandatory07_plus_F	TCGTCGGCAGCGTCAGATGTGTATAAGAGACAGGGTTAGAGGAGAYGTTTTAGTTTTT	59.2–60.9	
mandatory07_plus_R	GTCTCGTGGGCTCGGAGATGTGTATAAGAGACAGCAATTCCAAAAAACRTCAATCACAATAA	59.9–61.5	chr3:142837969-142838050
mandatory08_plus_F	TCGTCGGCAGCGTCAGATGTGTATAAGAGACAGGGTTAAGAGGAGTTTGTTTTGTTTTAT	60.8	
mandatory08_plus_R	GTCTCGTGGGCTCGGAGATGTGTATAAGAGACAGTTTCACTAAAAAACCTCACTCCCTA	60.9	chr7:140218100-140218192
mandatory09_plus_F	TCGTCGGCAGCGTCAGATGTGTATAAGAGACAGGTTTTAGAGTGTTTTTGGTTTTATTATTTTT	60.2	
mandatory09_plus_R	GTCTCGTGGGCTCGGAGATGTGTATAAGAGACAGTATTTACCCCTAAAAATACCCTTTATA	59.2	chr7:26206542-26206614
mandatory10_plus_F	TCGTCGGCAGCGTCAGATGTGTATAAGAGACAGGGAAGTTGAAGTGAGAATGTGATT	60.3	
mandatory10_plus_R	GTCTCGTGGGCTCGGAGATGTGTATAAGAGACAGAATACCCATACAAACTATCTACACAA	60.1	chr7:3025554-3025664
mandatory11_plus_F	TCGTCGGCAGCGTCAGATGTGTATAAGAGACAGTATATAAAAATTATTAAGAATTTTATTGTTTTGT	58.5	
mandatory11_plus_R	GTCTCGTGGGCTCGGAGATGTGTATAAGAGACAGAATATAACCAAAATCCAAATAACACTAA	58.2	chr7:138229946-138230021
mandatory12_plus_F	TCGTCGGCAGCGTCAGATGTGTATAAGAGACAGGYGGYGTTTGATGGATTTGGTTT	59.2–62.9	
mandatory12_plus_R	GTCTCGTGGGCTCGGAGATGTGTATAAGAGACAGCTTAATATAACCTAAACCCATATACTA	59.2	chr2:42275714-42275789
mandatory13_plus_F	TCGTCGGCAGCGTCAGATGTGTATAAGAGACAGGTAGATTATGTTAAGGATTTTGGAAAT	59.2	
mandatory13_plus_R	GTCTCGTGGGCTCGGAGATGTGTATAAGAGACAGTCTATACTATCAACACCCATTACTTAA	60.8	chr15:100249155-100249220
mandatory14_plus_F	TCGTCGGCAGCGTCAGATGTGTATAAGAGACAGTAAATTAGATGAGGTATAGTAGATTATAT	59.2	
mandatory14_plus_R	GTCTCGTGGGCTCGGAGATGTGTATAAGAGACAGCAACTCTATCTCAAACTTCAAAAAATA	59.2	chr4:147557821-147557938
mandatory15_plus_F	TCGTCGGCAGCGTCAGATGTGTATAAGAGACAGGTTGGGGGATAGTTTTGGGTAT	60.1	
mandatory15_plus_R	GTCTCGTGGGCTCGGAGATGTGTATAAGAGACAGTACAACCTCCTACAAAAAAACCCTA	60.9	chr17:75369174-75369252
mandatory16_plus_F	TCGTCGGCAGCGTCAGATGTGTATAAGAGACAGATATTTTTAATTTAATTTGAAGGTTTATTGT	57.8	
mandatory16_plus_R	GTCTCGTGGGCTCGGAGATGTGTATAAGAGACAGCCCAAACTTTCTCCTATAATCCAA	60.3	chr7:93520244-93520332
h19_plus_F	TCGTCGGCAGCGTCAGATGTGTATAAGAGACAGGTTTGTATTATTTTTTTTTTTGAGAGTTTATTT	60.2	
h19_plus_R	GTCTCGTGGGCTCGGAGATGTGTATAAGAGACAGATACRAAAAAAACCCACAATAAACTTAATA	59.8–61	chr11:2017873-2018050
mest_plus_F	TCGTCGGCAGCGTCAGATGTGTATAAGAGACAGGGTTTTGTTTTTTTAATTGTGTTTATTGTTT	60.2	
mest_plus_R	GTCTCGTGGGCTCGGAGATGTGTATAAGAGACAGTAACCACTATAACCAAAATTACACAAAA	59.9	chr7:130131098-130131299
xist_plus_F	TCGTCGGCAGCGTCAGATGTGTATAAGAGACAGGTAGTAATTTAGTATTGTTTATTTTATTTTTTT	59	
xist_plus_R	GTCTCGTGGGCTCGGAGATGTGTATAAGAGACAGATAACRAACCTCTTTATCTTTACTATATA	59.2–60.5	chrX:73070975-73071183
runx3_plus_F	TCGTCGGCAGCGTCAGATGTGTATAAGAGACAGTTTAGAYGTTYGGAGTTTTAGGGT	58.3–62	
runx3_plus_R	GTCTCGTGGGCTCGGAGATGTGTATAAGAGACAGCRACAACCCCAACTTCCTCTA	59.5–61.2	chr1:25256022-25256153
rarb_plus_F	TCGTCGGCAGCGTCAGATGTGTATAAGAGACAGAATTTTTTTATGYGAGTTGTTTGAGGAT	59.9–61.5	
rarb_plus_R	GTCTCGTGGGCTCGGAGATGTGTATAAGAGACAGCTCCTTCCAAATAAATACTTACAAAAAA	59.9	chr3:25469822-25469959
mlh1_plus_F	TCGTCGGCAGCGTCAGATGTGTATAAGAGACAGYGGGAGGTTATAAGAGTAGGGTT	60.9–62.9	
mlh1_plus_R	GTCTCGTGGGCTCGGAGATGTGTATAAGAGACAGATACRAAATATCCAACCAATAAAAACAAAA	59.8–61	chr3:37034573-37034734
rassf1a_plus_F	TCGTCGGCAGCGTCAGATGTGTATAAGAGACAGGTTTTYGTAGTTTAATGAGTTTAGGTTTT	60.5–62.1	
rassf1a_plus_R	GTCTCGTGGGCTCGGAGATGTGTATAAGAGACAGAATCCCTACACCCAAATTTCCATTA	60.9	chr3:50378200-50378398
apc_plus_F	TCGTCGGCAGCGTCAGATGTGTATAAGAGACAGGAGAGAGAAGTAGTTGTGTAAT	60.3	
apc_plus_R	GTCTCGTGGGCTCGGAGATGTGTATAAGAGACAGCATTCTATCTCCAATAACACCCTAA	60.9	chr5:112073447-112073596
cdkn2a_plus_F	TCGTCGGCAGCGTCAGATGTGTATAAGAGACAGATTTTGTTTTTTAAATTTTTTGGAGGGAT	59.2	
cdkn2a_plus_R	GTCTCGTGGGCTCGGAGATGTGTATAAGAGACAGCCCAACCTAAAACRACTTCAAAAATA	60.1–61.7	chr9:21974960-21975097
dapk1_p1_plus_F	TCGTCGGCAGCGTCAGATGTGTATAAGAGACAGTTTYGGAGTGTGAGGAGGATAGT	60.9–62.9	
dapk1_p1_plus_R	GTCTCGTGGGCTCGGAGATGTGTATAAGAGACAGRACRACRAAAACACAACTAAAAAATAAATA	58.5–62.6	chr9:90112783-90112938
dapk1_p2_plus_F	TCGTCGGCAGCGTCAGATGTGTATAAGAGACAGYGGAGGGATYGGGGAGTTTTT	62.1–65.5	
dapk1_p2_plus_R	GTCTCGTGGGCTCGGAGATGTGTATAAGAGACAGCCRCCTTAACCTTCCCAATTA	63.6–65.2	chr9:90112991-90113144
dapk1_i1_plus_F	TCGTCGGCAGCGTCAGATGTGTATAAGAGACAGGGAGGYGGGGAGGTTAGTTAT	61.2–63.2	
dapk1_i1_plus_R	GTCTCGTGGGCTCGGAGATGTGTATAAGAGACAGAAATAAAAAAAAACACCCTTTATTAAAACTAA	59.8	chr9:90113588-90113759
gstp1_plus_F	TCGTCGGCAGCGTCAGATGTGTATAAGAGACAGTTTGGGAAAGAGGGAAAGGTTTTT	60.3	
gstp1_plus_R	GTCTCGTGGGCTCGGAGATGTGTATAAGAGACAGRCRACCTCCRAACCTTATAAAAATAA	58.4–62.9	chr11:67351064-67351273
cdh1_snp_plus_F	TCGTCGGCAGCGTCAGATGTGTATAAGAGACAGATTTTAGTAATTTTAGGTTAGAGGGTT	59.2	
cdh1_snp_plus_R	GTCTCGTGGGCTCGGAGATGTGTATAAGAGACAGAAAAATAAATACRTAACTACAACCAAATAAA	59–60.2	chr16:68771006-68771197
cdh1_3ê_plus_F	TCGTCGGCAGCGTCAGATGTGTATAAGAGACAGGTYGGAATTGTAAAGTATTTGTGAGT	60.1–61.7	
cdh1_3ê_plus_R	GTCTCGTGGGCTCGGAGATGTGTATAAGAGACAGATCAAAAAATCCRAAATACCTACAACAA	59.5–61.5	chr16:68771201-68771385
brca1_plus_F	TCGTCGGCAGCGTCAGATGTGTATAAGAGACAGTTTAGTTATTTGAGAAATTTTATAGTTTGTT	59	
brca1_plus_R	GTCTCGTGGGCTCGGAGATGTGTATAAGAGACAGAATTTCRTATTCTAAAAAACTACTACTTAA	58.5–59.8	chr17:41277330-41277493
AluSx_1_plus_F	TCGTCGGCAGCGTCAGATGTGTATAAGAGACAGAGATTAGTTTGGTTAATATGGTGAAATT	59.9	
AluSx_1_plus_R	GTCTCGTGGGCTCGGAGATGTGTATAAGAGACAGCTCTATCRCCCAAACTAAAATACAATA	60.8–62.1	
AluSx_2_plus_F	TCGTCGGCAGCGTCAGATGTGTATAAGAGACAGGTTTGTAATTTTAGTATTTTGGGAGGT	60.8	
AluSx_2_plus_R	GTCTCGTGGGCTCGGAGATGTGTATAAGAGACAGAACCTCCCRAATAACTAAAACTACAA	60.1–61.7	
L1ME_ORF2_1_plus_F	TCGTCGGCAGCGTCAGATGTGTATAAGAGACAGATGATAAAAGGGTTAATTTATTAGAAAGAT	59.8	
L1ME_ORF2_1_plus_R	GTCTCGTGGGCTCGGAGATGTGTATAAGAGACAGCTATCTAATTATTCTRTCAATTACTAAAAA	58.5–59.8	
L1ME_ORF2_2_plus_F	TCGTCGGCAGCGTCAGATGTGTATAAGAGACAGGATTGATAAAGAAGAAAATAGATAAGATAT	59.8	
L1ME_ORF2_2_plus_R	GTCTCGTGGGCTCGGAGATGTGTATAAGAGACAGCTATTCAAATTTTCTATTTCTTTTTAAATCAA	59.8	
foxe3_2_F	TCGTCGGCAGCGTCAGATGTGTATAAGAGACAGTTTTGGGGAGGTTTATTTGAGGT	59.2	
foxe3_2_R	GTCTCGTGGGCTCGGAGATGTGTATAAGAGACAGAACRCAAAATATACTCCAAACCAAAATA	59.9–61.5	chr1
foxp3_1_plus_F	TCGTCGGCAGCGTCAGATGTGTATAAGAGACAGTTTGGGTTTAGGGTTTTATTTGTAGT	59.2	
foxp3_1_plus_R	GTCTCGTGGGCTCGGAGATGTGTATAAGAGACAGACCCAAAACCTCAAACCTACTAAA	60.3	chrX
foxp3_2_plus_F	TCGTCGGCAGCGTCAGATGTGTATAAGAGACAGTTTTTGGGGATGGGTTAAGGGTT	60.9	
foxp3_2_plus_R	GTCTCGTGGGCTCGGAGATGTGTATAAGAGACAGCAACCAATACCTACTTTAACCAAAAA	60.1	chrX
tlx3_1_plus_F	TCGTCGGCAGCGTCAGATGTGTATAAGAGACAGTTYGGTTTAAGAAAGATGATATAGAGTT	59.9–61.5	
tlx3_1_plus_R	GTCTCGTGGGCTCGGAGATGTGTATAAGAGACAGTCCATCCTAAACRAACRAAAAAACTAA	59.2–62.1	chr5
tlx3_2_plus_F	TCGTCGGCAGCGTCAGATGTGTATAAGAGACAGGGYGTTAGTTATTTGGGAGGGTTT	59.2–60.9	
tlx3_2_plus_R	GTCTCGTGGGCTCGGAGATGTGTATAAGAGACAGAACRCTAAACTCAAATTCACACTATAAA	59.5–61.5	chr5
uniq_noCG_1_plus_F	TCGTCGGCAGCGTCAGATGTGTATAAGAGACAGGAGTTATGTAGTTTTAGTTAGAAGTTT	59.2	
uniq_noCG_1_plus_R	GTCTCGTGGGCTCGGAGATGTGTATAAGAGACAGAAATCTAAATTTTAACACCTAAAACTATTTTAA	59.8	chr5
uniq_noCG_2_plus_F	TCGTCGGCAGCGTCAGATGTGTATAAGAGACAGATATGAAAGGTTGGTTTTATTGTTGAAT	59.9	
uniq_noCG_2_plus_R	GTCTCGTGGGCTCGGAGATGTGTATAAGAGACAGAAAATAAACTTAATAACTCTACTCTTATATA	59	chr5
mgmt_1_plus_F	TCGTCGGCAGCGTCAGATGTGTATAAGAGACAGGTTGAGTTAGGTTTTGGTAGTGTT	60.3	
mgmt_1_plus_R	GTCTCGTGGGCTCGGAGATGTGTATAAGAGACAGCTAATACCRCTCCCCTAATCAAAA	60.3–62	chr10
mgmt_2_plus_F	TCGTCGGCAGCGTCAGATGTGTATAAGAGACAGGTGGTAGTTTYGAGTGGTTTTGT	59.2–60.9	
mgmt_2_plus_R	GTCTCGTGGGCTCGGAGATGTGTATAAGAGACAGAACTAAACAACACCTAAAAAACACTTAA	59.9	chr10
mito_1_plus_F	TCGTCGGCAGCGTCAGATGTGTATAAGAGACAGTATTTATTTTTAATAGTATATAGTATATAAAGTT	58.5	
mito_1_plus_R	GTCTCGTGGGCTCGGAGATGTGTATAAGAGACAGACTTTAACTACCCCCAAATATTATAA	58.4	chrM
mito_2_plus_F	TCGTCGGCAGCGTCAGATGTGTATAAGAGACAGATGATTTTTAATAGGGGTTTTTTTAGTTT	59.2	
mito_2_plus_R	GTCTCGTGGGCTCGGAGATGTGTATAAGAGACAGCRTATCRAAAACCTTTTTAAACAAATAATA	58.5–61	chrM

Our initial QC assessment indicated high bisulfite conversion efficiency with very low non-CpG Cs in reads. An additional amplicon that corresponded to a sequence containing no CpG sites was also included as a control, from which all cytosines were observed to have converted to thymidine residues [1].

**Fig. 1:**
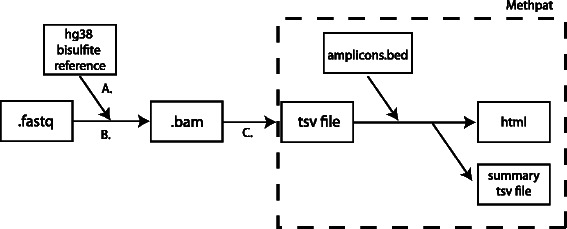
Flow of data towards visualization via Methpat. Raw fastq files are aligned to the hg38 reference genome in bisulfite space. **a** hg38 reference is prepared for Bismark using *Bismark_genome_preparation* with default parameters. **b** Bismark is used to align raw reads from fastq files to generate BAM alignment files. **c** *Bismark_methylation_extractor* is then used to extract the methylation status of all cytosines in every aligned read and outputs a tab-delimited file that Methpat operates on. Methpat requires this file along with a BED formatted file containing information for each amplicon of interest. This includes the start and end coordinates of the amplicon and the primer lengths for each amplicon. The output of Methpat is a summary tab-delimited file containing read counts of DNA methylation patterns of the amplicons of interest and an HTML file for visualization and publication quality figures

The data included here are the Sequence Read Archive files generated from our experiment. These have been aligned onto the hg38 reference genome using Bismark v0.9.0, from which a BAM file for each sample is generated. Using the *Bismark_methylation_extractor* command, the methylation status of cytosine residues within each read is output to a tab-delimited file. Methpat then operates on this output file to generate both a summarized tab-delimited file of read pattern counts and a HTML file for visualization. We have included the BAM files, *Bismark_methylation_extractor* output files and Methpat output files as supporting data. Methpat requires a Browser Extensive Data (BED)-format-like file that contains the coordinates for each amplicon of interest, their size and their primer lengths to extract and summarize DNA methylation pattern counts. The flow of data is summarized in Fig. 1.

Our data has the potential to be used to investigate co-methylation [8], given the unprecedented depth of coverage of the amplicons investigated even in a single MiSeq run. We have interrogated a variety of regions of the genome including repetitive elements and the mitochondrial genome, which remain a challenge for most short read aligners. The interpretation of DNA methylation at repetitive sequence elements has always been a challenge and they are assumed to be methylated [9]. However, the dynamics of repetitive element DNA methylation in cancer [10] and development [11] remain areas of interest that can now be properly interpreted with massively parallel sequencing and visualization tools such as Methpat.

## Availability of software and requirements

Project name: Methpat

Project home page: http://bjpop.github.io/methpat/

Operating system(s): any POSIX-like operating system (i.e.: Linux, OS X)

Programming language: Python 2.7, HTML and Javascript

Other requirements: Web Browser to view visualization output (HTML file). Suggested browsers include Firefox, Chrome or Safari. Methpat requires output files derived by Bismark (http://www.bioinformatics.babraham.ac.uk/projects/bismark/) and the *Bismark_methylation_extractor* command. Methpat can be accessed directly from http://bjpop.github.io/methpat/. With further instructions found at the URL.

License: 3-clause BSD License

Any restrictions to use by non-academics: None

A flow diagram of analytical requirements and files can be found in Fig. 1.

## Availability of supporting data and materials

Sequence files associated with main research publication deposited in GEO, GSE67856 [5]. Remaining files are deposited in GEO, GSE71804 [6].

BAM files, *bismark_methylation_extractor* output files and Methpat output files for each sample analyzed in this paper are available in the *GigaScience* GigaDB repository [12].

## Abbreviations

BED: Browser extensible data
RRBS: Reduced representation bisulfite sequencing
WGBS: Whole genome bisulfite sequencing
